# Methyl 5-chloro-4-hy­droxy-2,2-dioxo-1*H*-2λ^6^,1-benzo­thia­zine-3-carboxyl­ate: structure and Hirshfeld surface analysis

**DOI:** 10.1107/S2056989020012566

**Published:** 2020-09-18

**Authors:** Svitlana V. Shishkina, Lidiya A. Petrushova, Kateryna O. Burian, Andrii I. Fedosov, Igor V. Ukrainets

**Affiliations:** a SSI Institute for Single Crystals’ National Academy of Science of Ukraine, 60 Nauky ave., Kharkiv 61001, Ukraine; b Department of Inorganic Chemistry, V. N. Karazin Kharkiv National University, 4 Svobody Sq., Kharkiv 61077, Ukraine; c National University of Pharmacy, 4 Valentynivska St, Kharkiv 61168, Ukraine

**Keywords:** benzo­thia­zine derivative, mol­ecular structure, crystal structure, Hirshfeld surface analysis

## Abstract

The title compound, C_10_H_8_ClNO_5_S, which has potential analgesic activity, crystallizes in space group *P*2_1_/*n*. The benzo­thia­zine ring system adopts an inter­mediate form between sofa and twist-boat conformations. The coplanarity of the ester substituent to the bicyclic fragment is stabilized by an O—H⋯O intra­molecular hydrogen bond. In the crystal, hydrogen bonds of type N—H⋯O(SO_2_) link the mol­ecules into zigzag chains extending along the *b*-axis direction. Neighbouring chains are linked by both O—H⋯Cl and C—H⋯Cl inter­actions. A Hirshfeld surface analysis was used to compare different types of inter­molecular inter­actions, giving contributions of O⋯H/H⋯O = 42.0%, C⋯H/H⋯C = 17.3%, Cl⋯H/H⋯Cl = 14.2%, H⋯H = 11.1%.

## Abstract 2

The mol­ecular and crystal structure as well as the Hirshfeld surface were analysed for this benzo­thia­zine derivative with potential analgesic activity.

## Chemical context   

Alkyl 4-hy­droxy-2,2-dioxo-1*H*-2λ^6^,1-benzo­thia­zine-3-carbox­yl­ates are known to be highly active analgesics (Ukrainets *et al.*, 2013 ▸). The influence of substituents at the cyclic nitro­gen atom on the biological properties of these substances has been studied in detail (Ukrainets *et al.*, 2013 ▸, 2017 ▸). Continuing our research in this direction, we have synthesized and studied a new compound of this class with a substituent on the benzene part of the mol­ecule. The biological properties of benzo­thia­zine derivatives are known to depend on their mol­ecular structure (Ukrainets *et al.*, 2019*a* ▸,*b* ▸). In addition, such mol­ecules can form polymorphic modifications possessing different biological activity, as was shown in our previous studies (Ukrainets *et al.*, 2016*a* ▸, 2018 ▸). Therefore, the mol­ecular and crystal structure study as well as a Hirshfeld surface analysis were performed for the title compound, **1**.
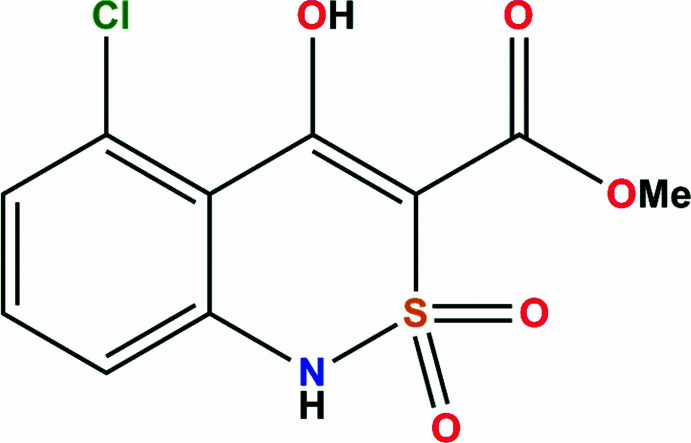


## Structural commentary   

**Figure 1:**
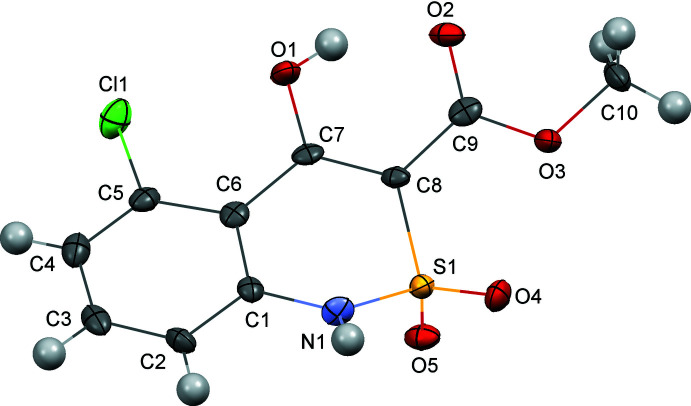
The mol­ecular structure of compound **1**. Displacement ellipsoids are drawn at the 50% probability level. Hydrogen atoms are shown as spheres of arbitrary radius.

The di­hydro­thia­zine ring in mol­ecule **1** adopts a conformation that is inter­mediate between a sofa and twist-boat (Fig. 1 ▸) with puckering parameters *S* = 0.53, *Θ* = 35.1°, *Ψ* = 11.3° (Zefirov *et al.*, 1990 ▸). The S1 and C8 atoms deviate by 0.81 (1) and 0.22 (1) Å, respectively, from the mean-square plane of the remaining atoms in the ring. The Cremer–Pople ring puckering parameters for the di­hydro­thia­zine ring are: *Q* = 0.457 (4) Å, *Θ* = 111.6 (5)°, *Ψ* = 192.1 (6)°. The ester substit­uent is essentially coplanar to the C7—C8 endocyclic double bond [the C7—C8—C9—O2 torsion angle is 3.0 (7)°] as a result of the stabilizing influence of the O1—H1*O*⋯O2 intra­molecular hydrogen bond (Table 1 ▸). This hydrogen bond can be specified as *S*(6) in terms of graph-set theory since the six atoms comprise a intra­molecular hydrogen-bonded motif. The formation of the O—H⋯O hydrogen bond causes some elongation of the C9—O2 and C7—C8 bonds as compared with typical values of 1.210 Å (C*sp* ^2^=O bond) and 1.326 Å (C*sp* ^2^—C*sp* ^2^ bond), respectively (Bürgi & Dunitz, 1994 ▸). The C7—O1 bond is shortened to 1.320 (6) Å (the typical length for a C*sp* ^2^—O bond is 1.362 Å) for the same reason. The methyl group is located in an *anti*-periplanar position to the C8—C9 bond [the C10—O3—C9—C8 torsion angle is −178.4 (4)°]. The noticeable steric repulsion between chlorine and the hydroxyl group [the Cl1⋯O1 distance is 2.793 (4) Å as compared to the van der Waals radii sum (Zefirov, 1997 ▸) of 3.19 Å] results in twisting of the Cl1—C5—C6—C7 and C5—C6—C7—O1 torsion angles up to 14.7 (7) and 14.5 (7)°, respectively.

## Supra­molecular features   

**Figure 2:**
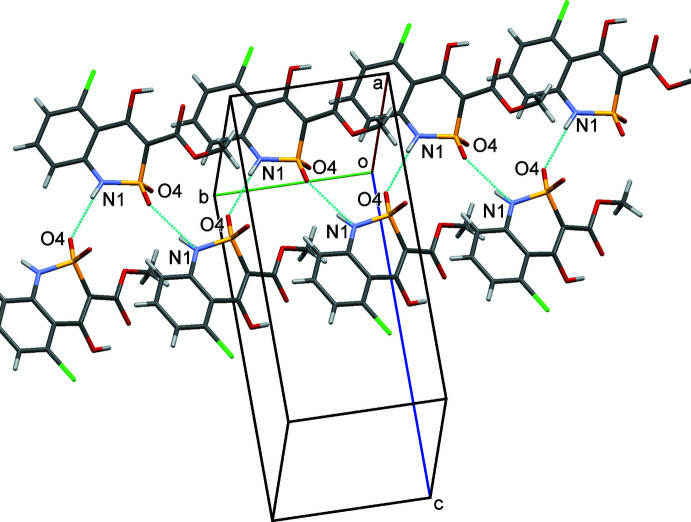
A chain of mol­ecules of **1** bound by N—H⋯O hydrogen bonds about the 2_1_-screw axis parallel to the *b* axis.

In the crystal, mol­ecules of **1** form zigzag chains in the [010] direction (Fig. 2 ▸) as a result of the formation of N1—H⋯O4^i^ hydrogen bonds about the 2_1_ screw axis parallel to *b* [symmetry code: (i) −*x* + 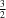, *y* + 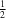, −*z* + 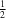; the N⋯O distance is 2.841 (5) Å, the N—H⋯O angle is 145.1° (Table 1 ▸)]. The crystal structure fragment formed by this hydrogen bond may be described as *C*(4) in terms of graph-set theory. Neighbouring zigzag chains are connected by weaker O—H⋯Cl^ii^ inter­actions (Table 1 ▸) in the [001] direction [symmetry code: (ii) 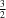 − *x*, *y* − 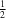, 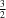 − *z*]. As a result, the hydrogen-bonded layers parallel to the *bc* plane may be considered as secondary structural motifs. There are weak C—H⋯Cl^iii^ inter­actions [symmetry code: (iii) *x* − 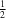, −*y* − 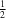, *z* − 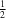] between mol­ecules of neighbouring layers. In addition, stacking inter­actions between inversion-related (2 − *x*, 1 − *y*, 1 − *z*) benzene rings of mol­ecules belonging to neighbouring layers are found. The distance between ring planes is 3.246 (2) Å. The stacking inter­actions are characterized by a centroid-to-centroid distance of 3.872 (2) Å, with a lateral shift of the benzene rings of 2.111 (2) Å.

## Hirshfeld surface analysis   

**Figure 3:**
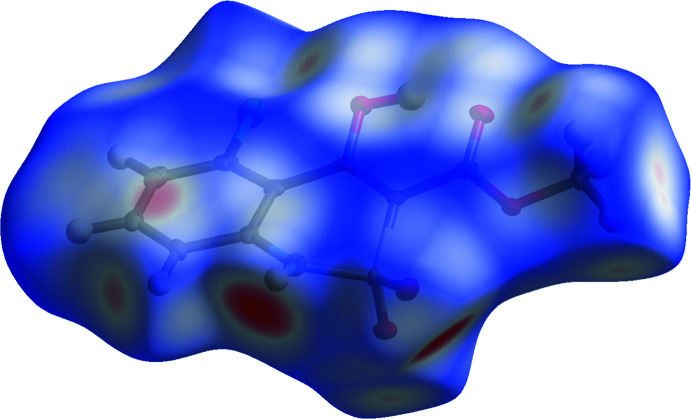
Hirshfeld surface of a mol­ecule of **1** mapped over *d*_norm_, with transparency to show the conformation.

Hirshfeld surface analysis and 2D fingerprint plots are useful tools to investigate the different types of intra- and inter­molecular inter­actions in a crystal (Turner *et al.*, 2017 ▸). The Hirshfeld surface of the title compound was obtained using a standard (high) surface resolution, mapped over *d*_norm_. The areas coloured red on the *d*_norm_ surfaces correspond to contacts that are shorter than the van der Waals radii sum of the closest atoms (Fig. 3 ▸). In this way, red spots on the Hirshfeld surface indicate atoms participating in hydrogen bonds or other short contacts. Such red spots are observed at the hydrogen atom of the NH group, one of the oxygen atoms of the SO_2_ group, and the chlorine atom. Smaller red spots are also present at one of the hydrogen atoms of the methyl group.

**Figure 4:**
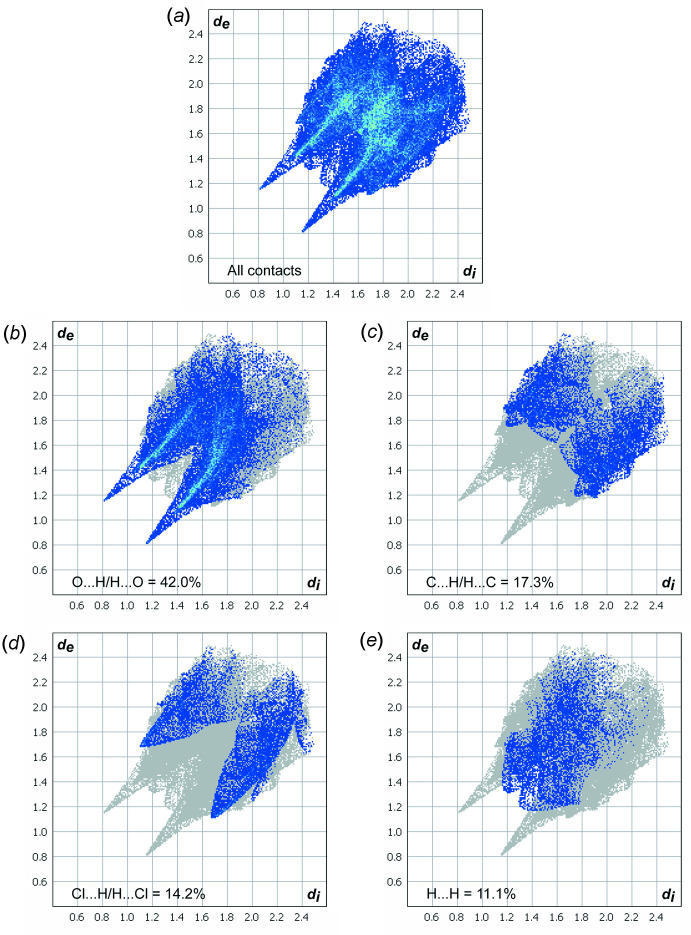
Two-dimensional Hirshfeld fingerprint plot of (*a*) all contacts for compound **1** and those delineated into (*b*) O⋯H/ H⋯O (42.0%), (*c*) C⋯H/H⋯C (17.3%), (*d*) Cl⋯H/H⋯Cl (14.2%), (*e*) H⋯H (11.1%) contacts.

All of the hydrogen bonds and short contacts of the title compound are evident in the two-dimensional fingerprint plot presented in Fig. 4 ▸ *a*. The pair of very sharp spikes indicates the presence of strong hydrogen bonds in the crystal of **1**. The main contribution (42.0%) to these spikes is provided by O⋯H/H⋯O inter­actions (Fig. 4 ▸ *b*). The contributions of C⋯H/H⋯C (17.3%) and Cl⋯H/H⋯Cl (14.2%) (Fig. 4 ▸ *c*,*d*) inter­actions are similar, but the presence of sharp spikes in the fingerprint plot delineated Cl⋯H/H⋯Cl inter­actions suggests that these contacts are stronger. Surprisingly, the contribution of H⋯H inter­actions (11.1%) (Fig. 4 ▸ *e*) is very small, which is unusual for mol­ecular crystals.

## Database survey   

A search of the Cambridge Structural Database (Version 5.41, update of November 2019; Groom *et al.*, 2016 ▸) for the benzo­thia­zine fragment revealed only ten hits [refcodes AKIJIP, AKIJIP01 (Ukrainets *et al.*, 2016*a* ▸), CABBEP (Lei *et al.*, 2016 ▸), IJUJAA (Ukrainets *et al.*, 2015*b* ▸), LANNUM (Ukrainets *et al.*, 2016*b* ▸), LOGHEW (Ukrainets *et al.*, 2014 ▸), MINJAW (Shishkina *et al.*, 2013 ▸), NODGUK (Ukrainets *et al.*, 2013 ▸), UWUCIA (Ukrainets *et al.*, 2015*a* ▸) and XEKPUB (Ukrainets *et al.*, 2017 ▸)]. In all these structures, the conformation of the benzo­thia­zine rings as well as the redistribution of the electron density as a result of the formation of the O—H⋯O intra­molecular hydrogen bond are similar.

The title compound may be considered as a structural analogue of methyl 4-hy­droxy-2,2-dioxo-1-methyl-1*H*-2,1-benzo­thia­zine-3-carboxyl­ate (Ukrainets *et al.*, 2013 ▸), which is substituted by chlorine on the benzene ring of the mol­ecule and de­alkyl­ated at the cyclic nitro­gen atom.

## Synthesis and crystallization   

**Figure 5:**
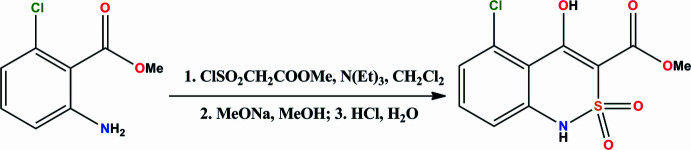
The synthesis of compound **1**.

Methyl (chloro­sulfon­yl)acetate (1.90 g, 0.011 mol) was added dropwise under stirring to a solution of methyl 6-chloro­anthranilate (1.85 g, 0.010 mol) and tri­ethyl­amine (1.54 mL, 0.011 mol) in CH_2_Cl_2_ (20 mL) and the mixture was cooled (268 to 273 K) (Fig. 5 ▸). After 10 h, water (50 mL) was added to the reaction mixture, which was acidified up to pH = 4 with 1 *N* HCl and mixed thoroughly. The organic layer was separated, dried over anhydrous CaCl_2_, and distilled (at reduced pressure at the end). A solution of sodium methyl­ate in anhydrous methanol [from metallic sodium (0.69 g, 0.030 mol) and absolute methanol (20 mL)] was added, the mixture was boiled and stored for 15 h at room temperature. The reaction mixture was diluted with cold water and acidified with 1 *N* HCl to pH = 4. The solid methyl 5-chloro-4-hy­droxy-2,2-dioxo-1*H*-2λ^6^,1-benzo­thia­zine-3-carboxyl­ate was filtered, washed with water, and dried in air. Yield 2.43g (84%); colourless crystals; m.p. 464–466 K.

## Refinement   

Crystal data, data collection and structure refinement details are summarized in Table 2 ▸. All of the hydrogen atoms were located in difference-Fourier maps. They were included in calculated positions and treated as riding with C—H = 0.96 Å, O—H = 0.84 Å, *U*_iso_(H) = 1.5*U*_eq_(C,O) for methyl and hydroxyl groups and with C—H = 0.93 Å, N—H = 0.88 Å, *U*_iso_(H) = 1.2*U*_eq_(C,N) for all other hydrogen atoms.

## Supplementary Material

Crystal structure: contains datablock(s) I. DOI: 10.1107/S2056989020012566/pk2647sup1.cif

Structure factors: contains datablock(s) I. DOI: 10.1107/S2056989020012566/pk2647Isup2.hkl

Supporting information file. DOI: 10.1107/S2056989020012566/pk2647Isup3.cml

CCDC reference: 2032151

Additional supporting information:  crystallographic information; 3D view; checkCIF report

## Figures and Tables

**Table 1:** Hydrogen-bond geometry (Å, °)

*D*—H⋯*A*	*D*—H	H⋯*A*	*D*⋯*A*	*D*—H⋯*A*
O1—H1*O*⋯O2	0.84	1.76	2.509 (5)	148
N1—H1*N*⋯O4^i^	0.88	2.07	2.841 (5)	145
O1—H1*O*⋯Cl1^ii^	0.84	2.87	3.203 (4)	106
C10—H10*C*⋯Cl1^iii^	0.98	2.84	3.399 (5)	117

Symmetry codes: (i) 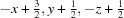; (ii) 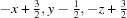; (iii) 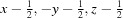.

**Table 2:** Experimental details

Crystal data	
Chemical formula	C_10_H_8_ClNO_5_S
*M*_r_	289.68
Crystal system, space group	Monoclinic, *P*2_1_/*n*
Temperature (K)	120
*a*, *b*, *c* (Å)	11.153 (3), 6.8926 (15), 14.600 (3)
β (°)	97.528 (5)
*V* (Å^3^)	1112.6 (4)
*Z*	4
Radiation type	Mo *K*α
μ (mm^−1^)	0.54
Crystal size (mm)	0.30 × 0.10 × 0.05

Data collection	
Diffractometer	Rigaku Oxford Diffraction Xcalibur, Sapphire3
Absorption correction	Multi-scan (*CrysAlis PRO*; Rigaku OD, 2018 ▸)
*T*_min_, *T*_max_	0.428, 1.000
No. of measured, independent and observed [*I* > 2σ(*I*)] reflections	9166, 1957, 1343
*R*_int_	0.111
(sin θ/λ)_max_ (Å^−1^)	0.594

Refinement	
*R*[*F* ^2^ > 2σ(*F* ^2^)], *wR*(*F* ^2^), *S*	0.063, 0.145, 1.05
No. of reflections	1957
No. of parameters	165
H-atom treatment	H-atom parameters constrained
Δρ_max_, Δρ_min_ (e Å^−3^)	0.38, −0.46

Computer programs: *CrysAlis PRO* (Rigaku OD, 2018 ▸), *SHELXT* (Sheldrick, 2015*a* ▸), *SHELXL* (Sheldrick, 2015*b* ▸) and *Mercury* (Macrae, 2020 ▸).

## supplementary crystallographic information

### Crystal data

**Table:**

C_10_H_8_ClNO_5_S	*F*(000) = 592
*M**_r_* = 289.68	*D*_x_ = 1.729 Mg m^−^^3^
Monoclinic, *P*2_1_/*n*	Mo *K*α radiation, λ = 0.71073 Å
*a* = 11.153 (3) Å	Cell parameters from 2736 reflections
*b* = 6.8926 (15) Å	θ = 3.3–34.2°
*c* = 14.600 (3) Å	µ = 0.54 mm^−^^1^
β = 97.528 (5)°	*T* = 120 K
*V* = 1112.6 (4) Å^3^	Plate, colourless
*Z* = 4	0.30 × 0.10 × 0.05 mm

### Data collection

**Table:**

Rigaku Oxford Diffraction Xcalibur, Sapphire3 diffractometer	1957 independent reflections
Radiation source: Enhance (Mo) X-ray Source	1343 reflections with *I* > 2σ(*I*)
Detector resolution: 16.1827 pixels mm^-1^	*R*_int_ = 0.111
ω scans	θ_max_ = 25.0°, θ_min_ = 2.2°
Absorption correction: multi-scan (CrysAlisPro; Rigaku OD, 2018)	*h* = −13→13
*T*_min_ = 0.428, *T*_max_ = 1.000	*k* = −8→8
9166 measured reflections	*l* = −17→17

### Refinement

**Table:**

Refinement on *F*^2^	0 restraints
Least-squares matrix: full	Hydrogen site location: difference Fourier map
*R*[*F*^2^ > 2σ(*F*^2^)] = 0.063	H-atom parameters constrained
*wR*(*F*^2^) = 0.145	*w* = 1/[σ^2^(*F*_o_^2^) + (0.0609*P*)^2^ + 0.6529*P*] where *P* = (*F*_o_^2^ + 2*F*_c_^2^)/3
*S* = 1.05	(Δ/σ)_max_ < 0.001
1957 reflections	Δρ_max_ = 0.38 e Å^−^^3^
165 parameters	Δρ_min_ = −0.46 e Å^−^^3^

### Special details

**Table:**

Geometry. All esds (except the esd in the dihedral angle between two l.s. planes) are estimated using the full covariance matrix. The cell esds are taken into account individually in the estimation of esds in distances, angles and torsion angles; correlations between esds in cell parameters are only used when they are defined by crystal symmetry. An approximate (isotropic) treatment of cell esds is used for estimating esds involving l.s. planes.

### Fractional atomic coordinates and isotropic or equivalent isotropic displacement parameters (Å^2^)

**Table:**

	*x*	*y*	*z*	*U*_iso_*/*U*_eq_	
Cl1	0.84549 (13)	0.2458 (2)	0.73322 (9)	0.0298 (4)	
S1	0.76398 (12)	0.09884 (18)	0.35643 (9)	0.0158 (3)	
O1	0.6753 (3)	0.0566 (5)	0.6066 (2)	0.0218 (9)	
H1O	0.638729	−0.048715	0.594436	0.033*	
O2	0.5710 (3)	−0.2111 (5)	0.5105 (2)	0.0232 (9)	
O3	0.6006 (3)	−0.2377 (5)	0.3617 (2)	0.0212 (9)	
O4	0.6887 (3)	0.0811 (5)	0.2691 (2)	0.0209 (8)	
O5	0.8815 (3)	0.0129 (5)	0.3625 (2)	0.0213 (9)	
N1	0.7714 (4)	0.3285 (6)	0.3778 (3)	0.0200 (10)	
H1N	0.750045	0.409629	0.331963	0.024*	
C1	0.8100 (4)	0.4052 (7)	0.4653 (3)	0.0150 (11)	
C2	0.8599 (4)	0.5923 (7)	0.4729 (4)	0.0187 (12)	
H2	0.867292	0.666061	0.418938	0.022*	
C3	0.8974 (4)	0.6672 (8)	0.5574 (4)	0.0215 (12)	
H3	0.928646	0.795566	0.562115	0.026*	
C4	0.8914 (5)	0.5596 (8)	0.6383 (4)	0.0225 (13)	
H4	0.921192	0.611991	0.697051	0.027*	
C5	0.8413 (4)	0.3765 (8)	0.6308 (3)	0.0182 (12)	
C6	0.7937 (4)	0.2947 (7)	0.5441 (3)	0.0156 (11)	
C7	0.7202 (4)	0.1186 (7)	0.5325 (3)	0.0163 (11)	
C8	0.6938 (4)	0.0228 (7)	0.4494 (3)	0.0152 (11)	
C9	0.6170 (4)	−0.1515 (7)	0.4434 (3)	0.0180 (12)	
C10	0.5228 (5)	−0.4067 (7)	0.3536 (4)	0.0247 (13)	
H10C	0.528740	−0.471978	0.294700	0.037*	
H10B	0.438987	−0.366365	0.355781	0.037*	
H10A	0.548102	−0.495994	0.404669	0.037*	

### Atomic displacement parameters (Å^2^)

**Table:**

	*U*^11^	*U*^22^	*U*^33^	*U*^12^	*U*^13^	*U*^23^
Cl1	0.0396 (9)	0.0322 (8)	0.0162 (7)	0.0041 (7)	−0.0016 (6)	0.0010 (6)
S1	0.0175 (7)	0.0156 (7)	0.0151 (7)	−0.0009 (6)	0.0050 (5)	−0.0007 (6)
O1	0.030 (2)	0.020 (2)	0.0181 (19)	−0.0045 (17)	0.0113 (17)	0.0001 (16)
O2	0.022 (2)	0.023 (2)	0.028 (2)	−0.0011 (17)	0.0127 (17)	0.0031 (17)
O3	0.023 (2)	0.021 (2)	0.022 (2)	−0.0081 (17)	0.0110 (16)	−0.0049 (16)
O4	0.023 (2)	0.025 (2)	0.0143 (18)	0.0003 (17)	−0.0004 (15)	−0.0031 (16)
O5	0.020 (2)	0.019 (2)	0.028 (2)	0.0041 (16)	0.0107 (16)	0.0025 (16)
N1	0.029 (3)	0.014 (2)	0.017 (2)	0.003 (2)	0.004 (2)	0.0069 (18)
C1	0.009 (2)	0.017 (3)	0.019 (3)	0.002 (2)	0.001 (2)	−0.002 (2)
C2	0.017 (3)	0.013 (3)	0.027 (3)	−0.002 (2)	0.005 (2)	0.001 (2)
C3	0.011 (3)	0.018 (3)	0.036 (3)	0.002 (2)	0.005 (2)	−0.008 (2)
C4	0.015 (3)	0.028 (3)	0.024 (3)	0.005 (2)	−0.001 (2)	−0.008 (2)
C5	0.011 (3)	0.027 (3)	0.017 (3)	0.003 (2)	0.003 (2)	0.000 (2)
C6	0.010 (3)	0.018 (3)	0.019 (3)	0.006 (2)	0.002 (2)	0.000 (2)
C7	0.010 (3)	0.022 (3)	0.018 (3)	0.005 (2)	0.006 (2)	0.002 (2)
C8	0.013 (3)	0.014 (3)	0.019 (3)	0.002 (2)	0.007 (2)	0.001 (2)
C9	0.014 (3)	0.020 (3)	0.020 (3)	0.010 (2)	0.003 (2)	0.003 (2)
C10	0.020 (3)	0.021 (3)	0.034 (3)	−0.008 (3)	0.007 (2)	−0.009 (3)

### Geometric parameters (Å, º)

**Table:**

Cl1—C5	1.741 (5)	C1—C2	1.403 (7)
S1—O5	1.431 (4)	C1—C6	1.412 (7)
S1—O4	1.437 (3)	C2—C3	1.352 (7)
S1—N1	1.614 (4)	C3—C4	1.404 (7)
S1—C8	1.734 (5)	C4—C5	1.379 (7)
O1—C7	1.320 (6)	C5—C6	1.423 (7)
O2—C9	1.234 (6)	C6—C7	1.462 (7)
O3—C9	1.323 (6)	C7—C8	1.379 (7)
O3—C10	1.448 (6)	C8—C9	1.471 (7)
N1—C1	1.397 (6)		
			
O5—S1—O4	116.3 (2)	C4—C5—Cl1	116.2 (4)
O5—S1—N1	111.8 (2)	C6—C5—Cl1	121.6 (4)
O4—S1—N1	105.3 (2)	C1—C6—C5	116.0 (5)
O5—S1—C8	109.3 (2)	C1—C6—C7	118.9 (4)
O4—S1—C8	113.4 (2)	C5—C6—C7	124.8 (4)
N1—S1—C8	99.3 (2)	O1—C7—C8	120.3 (5)
C9—O3—C10	116.5 (4)	O1—C7—C6	116.2 (4)
C1—N1—S1	123.3 (3)	C8—C7—C6	123.5 (4)
N1—C1—C2	119.5 (4)	C7—C8—C9	119.9 (4)
N1—C1—C6	119.0 (4)	C7—C8—S1	118.5 (4)
C2—C1—C6	121.5 (5)	C9—C8—S1	121.3 (4)
C3—C2—C1	119.7 (5)	O2—C9—O3	122.9 (5)
C2—C3—C4	121.5 (5)	O2—C9—C8	121.6 (5)
C5—C4—C3	118.8 (5)	O3—C9—C8	115.5 (4)
C4—C5—C6	122.2 (5)		
			
O5—S1—N1—C1	−69.3 (4)	C5—C6—C7—O1	14.5 (7)
O4—S1—N1—C1	163.5 (4)	C1—C6—C7—C8	19.0 (7)
C8—S1—N1—C1	45.9 (4)	C5—C6—C7—C8	−167.7 (5)
S1—N1—C1—C2	154.5 (4)	O1—C7—C8—C9	−1.2 (7)
S1—N1—C1—C6	−27.8 (6)	C6—C7—C8—C9	−178.8 (4)
N1—C1—C2—C3	−180.0 (4)	O1—C7—C8—S1	−175.2 (3)
C6—C1—C2—C3	2.3 (7)	C6—C7—C8—S1	7.2 (7)
C1—C2—C3—C4	2.1 (7)	O5—S1—C8—C7	82.9 (4)
C2—C3—C4—C5	−2.7 (7)	O4—S1—C8—C7	−145.5 (4)
C3—C4—C5—C6	−1.2 (7)	N1—S1—C8—C7	−34.2 (4)
C3—C4—C5—Cl1	176.1 (4)	O5—S1—C8—C9	−91.1 (4)
N1—C1—C6—C5	176.5 (4)	O4—S1—C8—C9	40.5 (5)
C2—C1—C6—C5	−5.8 (7)	N1—S1—C8—C9	151.8 (4)
N1—C1—C6—C7	−9.7 (7)	C10—O3—C9—O2	1.1 (7)
C2—C1—C6—C7	168.0 (4)	C10—O3—C9—C8	−178.4 (4)
C4—C5—C6—C1	5.2 (7)	C7—C8—C9—O2	3.0 (7)
Cl1—C5—C6—C1	−171.9 (4)	S1—C8—C9—O2	176.9 (4)
C4—C5—C6—C7	−168.2 (5)	C7—C8—C9—O3	−177.5 (4)
Cl1—C5—C6—C7	14.7 (7)	S1—C8—C9—O3	−3.6 (6)
C1—C6—C7—O1	−158.7 (4)		

### Hydrogen-bond geometry (Å, º)

**Table:**

*D*—H···*A*	*D*—H	H···*A*	*D*···*A*	*D*—H···*A*
O1—H1*O*···O2	0.84	1.76	2.509 (5)	148
N1—H1*N*···O4^i^	0.88	2.07	2.841 (5)	145
O1—H1*O*···Cl1^ii^	0.84	2.87	3.203 (4)	106
C10—H10*C*···Cl1^iii^	0.98	2.84	3.399 (5)	117

Symmetry codes: (i) −*x*+3/2, *y*+1/2, −*z*+1/2; (ii) −*x*+3/2, *y*−1/2, −*z*+3/2; (iii) *x*−1/2, −*y*−1/2, *z*−1/2.
